# Feedback in clinical settings: Nursing students’ perceptions at the district hospital in the southern part of Namibia

**DOI:** 10.4102/curationis.v44i1.2147

**Published:** 2021-05-31

**Authors:** Vistolina Nuuyoma

**Affiliations:** 1 School of Nursing, Faculty of Health Science, University of Namibia, Rundu, Namibia

**Keywords:** clinical education, clinical learning, clinical practice, clinical settings, clinical teaching, feedback

## Abstract

**Background:**

Feedback was the backbone of educational interventions in clinical settings. However, it was generally misunderstood and demanding to convey out effectively. Nursing students were not confident and did not feel free to practise clinical skills during practical placements because of the nature of the feedback they received whilst in these placements. Moreover, they experienced feedback as a barrier to completing practical workbooks.

**Objective:**

The purpose of this article was to report on a qualitative study, which explored nursing students’ perceptions of the feedback they received in clinical settings, at a district hospital.

**Method:**

This study was conducted at a district hospital located in southern Namibia. An explorative qualitative design with an interpretivist perspective was followed. A total of 11 nursing students from two training institutions were recruited by purposive sampling and were interviewed individually. All interviews were audio recorded with a digital voice recorder followed by verbatim transcriptions, with the participants’ permission. Thereafter, data were analysed manually by qualitative content analysis.

**Results:**

Themes that emerged as findings of this study are feedback is perceived as a teaching and learning process in clinical settings; participants perceived the different nature of feedback in clinical settings; participants perceived personal and interpersonal implications of feedback and there were strategies to improve feedback in clinical settings.

**Conclusion:**

Nursing students appreciated the feedback they received in clinical settings, despite the challenges related to group feedback and the emotional reactions it provoked. Nursing students should be prepared to be more receptive to the feedback conveyed in clinical settings.

## Introduction

Feedback refers to a practice in which leaners make sense of remarks about the value of their works for the purpose of future development in performance or learning strategies (Carless 2019:705–714). It is one of the basic elements that should be present in educational strategies utilised during clinical practice. In order for nursing students to learn clinical skills, key individuals within each clinical unit support them to identify learning opportunities. Therefore, clinical practices are considered fundamental for skills acquisition during the training of nursing students, where competencies for personal and professional development are observed, include leadership, interpersonal and communication skills (González-García et al. 2020:1–14). These lead nursing students to become capable, competent and caring nurses (Dasila et al. 2016:37–41). Nursing students must then make sense of their clinical practice through the application of theory into practice, reflection on their experience and feedback. Feedback assists nursing students to think about the gap between real and anticipated performance and find methods to reduce the gap and improve on it. More importantly, it stimulates reflective and experiential learning and encourages nursing students to reflect on feelings, experiences and incidents (Hardavella et al. 2017:327–333). Although feedback is widely acknowledged as an important element in clinical education, it is a component in which educators continue to fall short (Weinstein 2015:559–561). Al-Bashir, Kabir and Rahman (2016:38–41) reported that feedback is considered as a challenging matter in the higher education arena. Moreover, feedback in higher education is generally misunderstood and must be conveyed effectively (Carless & Boud 2018:1315–1325).

Nursing education routinely employs a scaffold approach in the curricula, whereby information is presented by continually building on each other, throughout the programme of study (Pront & McNeill 2019:85–90). This is made through the theoretical and practical components. The theoretical component is usually taught at the training institution premises or via online teaching using learning management systems. The practical component is taught via clinical practice in clinical settings. Clinical settings are multifaceted environments with a combination of social, institutional and political structures (Dobrowolska et al. 2015:36–46). Dominantly, training in clinical settings employs a dyadic approach that consists of the clinical environment itself and supervisory relationships between nursing students and educators (Rajeswaran 2017:1–6). For successful implementation of clinical practice, it requires components such as teaching aids, students, hospital staff, training institution staff, patients/clients, financial resources, clinical settings and facilities. The hospital and training institution staff are the backbone of the clinical practice support system, who play significant roles in the facilitation of learning and assessment of students, whereby provision of feedback is the key element. This implies that feedback is one of the supportive systems in place to assist in the facilitation of nursing students’ proficient development (Kalyani et al. 2019:1–8). However, for feedback to be of value, observing nursing students whilst in clinical practice is a pre-requisite (Burgess & Mellis 2015:373–381). Feedback entails delivering information to students with the purpose of reducing the discrepancy between their current and desired performance (Alfehaid et al. 2018:186–197). According to Burgess and Mellis (2015:373–381), the provision of feedback to students in clinical practice offers a valuable method of enriching the students’ learning experiences. If nursing students do not receive feedback, they may assume that everything is acceptable and will continue performing in the same way. This leads to wrong judgement of their own skills and abilities and creates a false perception (Hardavella et al. 2017:327–333). Therefore, clinical educators are encouraged to provide continuous feedback to students about their performance and how they can improve on it (Gaberson, Oermann & Shellenbarger 2015:64).

The nature of feedback received and provided to students in clinical settings includes formal, informal, written and oral/verbal feedback (Fowler & Wilford 2016:16–24). In addition, students also receive directive, facilitative and constructive feedback. The informal feedback highlights key aspects of the observed performance without deeper details and it is valuable in the sense that it only focuses on the significant part of the students’ actions. The formal feedback is more detailed, primarily in written form. In clinical settings, the latter is usually given during the mid-term or at the end of clinical practice period, which generally forms part of the work-based assessment (Johnson et al. 2019:1–11). Feedback is said to be directive when it enlightens the student about adjustments to be made. Conversely, it is facilitative when commentaries or suggestions are for students to do their own revision and improvements (Sultan & Khan 2017:1078–1084). Feedback is constructive when it is detailed, descriptive, well planned and conveyed in an appropriate interpersonal encounter. Additionally, it should be expected by the receiver, based on direct observation of an activity and focused on students’ performance rather than generalised comments (Sultan & Khan 2017:1078–1084).

During clinical practice, mentors and clinical instructors may ask patients to provide feedback to the students, especially to rate the level of compassion, respectfulness, commitment, treating others with dignity and ability to maintain confidentiality (Houghton 2016:41–49). Although students are a little hesitant to provide feedback on behaviours of peers and find it difficult to balance constructive and positive feedback, peer feedback is widely used in clinical settings, especially after peer observations (Pedram et al. 2020:1–10). It is therefore evident that feedback in clinical settings is not only given by clinical educators but peers and patients are also involved.

Previous studies reported nursing students’ perceptions of feedback. In a study conducted in Saudi Arabia to evaluate undergraduate health science students’ perceptions and attitudes of feedback, where nursing students were included, findings revealed that there are barriers to the provision of constructive feedback such as busy schedules, lack of communication skills amongst feedback providers and a large number of students. In addition, health science students were exposed to negative feedback practices such as comparing students, nonstandardised and irrelevant feedback (Alfehaid et al. 2018:373–381). Moreover, health science students perceived feedback given in clinical settings as too generic and ill-timed (Fowler & Wilford 2016:16–24). This was despite the common practice of advising feedback providers to convey feedback as close to the incident or exposure and to be specific as possible. Because students work with various mentors and supervisors, it hinders the provision of constructive and meaningful feedback (Fowler & Wilford 2016:16–24). Allen and Molloy (2017:57–62) reported that nursing students viewed feedback in clinical education as a valuable tool that motivates better performance. Moreover, appropriate feedback is supportive when it comes unexpectedly and is commenced by their preceptors. However, the provision of patient care limits feedback time in clinical environments as it is considered a priority in the ward routine. In clinical settings, feedback is seen by nursing students and educators as a shared responsibility, which involves asking, acting and receiving feedback from the complex team of all healthcare professionals (Adamson et al. 2018:48–53). This means that the healthcare team (including students) should not keep waiting to receive feedback but should actively seek and act on it. Walsh, Anstey and Tracey (2018:10–16) reported that students were given opportunities to explain and express their thoughts and feelings about events they encountered. The feedback given mostly focused on patient safety and strategies to prevent errors in future. As far as location and privacy are concerned, feedback was given in private settings, not visible to other students or patients. Although their study focused on nursing students’ perspectives on feedback given after medication errors, it reflects constructive and student-centred feedback conveyed to students whilst in clinical settings.

When used effectually, feedback can be used to adjust learning and teaching activities to meet the demands of the students (Mcfadzien 2015:16–18). Feedback is effective if students act on it to advance in their future work and learning situations. In the Keetmanshoop District, Namibia, which is the context for this study, students are nurses at different levels of their studies, and feedback providers are registered and enrolled nurses and midwives from clinical settings. In addition to the clinical instructors, there are also tutors and lecturers from the health training institutions. The staff from training institutions such as universities and nursing colleges are also considered as feedback providers in the context of this study.

### Problem statement

Irrespective of the significance of feedback in clinical practice, many health science students, including nurses, are disgruntled with the feedback they receive (Alfehaid et al. 2018:373–381). The predominant complaint is that they are not given adequate feedback (Burgess & Mellis 2015:373–381). Similarly, in the Keetmanshoop District, some nursing students were not confident and did not feel free to practise their nursing skills during their practical placements because of the nature of the feedback they received whilst in these placements. This was reported at student nurse-lecturer forums, which are platforms for nursing students and lecturers to meet regularly to discuss general academic and non-academic issues affecting their training. Furthermore, informal conversations with nursing students in the Keetmanshoop District indicated that the feedback they received was experienced as a barrier to completing their practical workbooks. The feedback did not help them improve and complete their practical registers as it was too general, did not reflect on their positive performances and was not focused on the task at hand. This was a barrier because they were not motivated to learn in clinical settings as they were unsure of how to improve their performance. Students do not find feedback useful in learning practical skills when it only focuses on bad performances (Abraham & Singaram 2016:121–125). To date, there is no evidence of a study being conducted that explored students’ experiences and perceptions of feedback in clinical settings. Therefore, their perceptions on feedback remain unknown. This led to the formulation of the following research question: *How do nursing students in the Keetmanshoop District perceive the feedback they receive in clinical settings*?

### Purpose of the study

The purpose of the article is to report on a qualitative study that explored nursing students’ perceptions of the feedback they received in clinical settings at a district hospital.

## Design and methods

### Research design

An explorative, qualitative research design with an interpretivist perspective was used in this study. The explorative qualitative approach was useful for exploring and understanding students’ perceptions of feedback, which was the central phenomenon under study (Creswell 2014a:55). The interpretive perspective was added to understand feedback through the meaning that research participants assigned to their daily clinical practice (Polit & Beck 2017:506).

### Research setting

The study was conducted at a district hospital, a 154-bed hospital located in the Keetmanshoop District, Southern Namibia. The hospital consists of a maternity unit, female, male, paediatric and tuberculosis (TB) wards, operating theatres, an outpatient department and a casualty department. There are two higher education institutions in the region that places nursing students at the district hospital. The students are placed for clinical practice for a duration of 2–4 weeks in one unit before being shifted to another unit. In all nursing programmes offered in Namibia, students have practical workbooks for each course with a practical component. These workbooks list the clinical learning experiences that students have to undergo and a space is provided for the registered nurse to sign after the demonstration of competency in a specific skill. The demonstration of competency from a student is observed through daily practice and conducting procedures whereby the nurse educator uses a checklist to rate performance after direct observation. In both cases, students are expected to receive feedback on their performance, conveyed to the student upon completion of a procedure or at the end of the shift. This implies that students should receive feedback to rate their performances against the expected standards. In addition, there are also a minimum number of procedures that learners should complete for each study level. These also serve as requirements for registration by the Health Professional Council of Namibia upon completion of the programme. Therefore, it is compulsory for all learners to complete their workbooks before they proceed to the next level of study.

### Population and sampling

There were a total of 82 students who practised in the Keetmanshoop district hospital during the 2016 academic year. A total of 11 nursing students were interviewed for the study being reported on, determined by data saturation. Of the 11 nursing students who participated, five were from the Bachelor of Nursing Science programme, three from the Diploma in Nursing Science and three were Certificate in Nursing Science students. Seven were female and four were male students. In accordance with qualitative research, this study employed non-probability and purposive sampling (Maree 2016:198). The criteria used to select participants stipulated that they should be in their second, third or fourth year of study (first-year clinical placements commenced only in May 2016) and had to be doing clinical practice in the Keetmanshoop District.

### Data collection methods

The data gathering technique used in this study was one-on-one in-depth interviews. This was made by interviewing participants face to face, individually to allow eliciting of perceptions on feedback in clinical settings (Creswell 2014b:190). The prospective participants were contacted personally in the lecture halls on campus and some in the clinical settings. The researcher explained the purpose of the study and gave the students a copy of the participant information sheet, together with a consent form. A follow-up was performed the next day to enquire whether the students agreed to participate in the study. All participants signed the informed consent forms prior to the interviews. An interview schedule was then drawn up according to the availability of the students and the interviewer. Interviews took place at the staff offices located at the two training institutions. The duration of the interviews ranged from 28 min to 37 min. Participants were asked to respond to the following central question: *how do you perceive feedback given to nursing students in clinical settings at the district hospital?* Moreover, prompts were made to explore their perceptions on the nature of feedback, positive and negative aspects and suggestions for improvements of feedback amongst nursing students in clinical settings. The one-on-one interviews were concluded when all the issues in the interview guide had been addressed and the participants and researcher had nothing to add. All interviews were conducted by the principal researcher, who was a lecturer at one of the training institutions located in the Keetmanshoop district at the time the study was conducted. The researcher taught a theory module with no practical component and was therefore not involved in clinical follow-ups of nursing students; hence, she was not involved in the provision of feedback in clinical settings. The two co-researchers did not participate in data collection, but audio recordings and transcriptions were shared with them, as they played a supervisory role in the research project.

All interviews were conducted in English, which is an official language in Namibia, and all participants indicated that they were comfortable to express themselves. Permission to audio record and transcribe was requested and granted in writing prior to the beginning of each interview. Audio recording was made to ensure that data reflected participants’ actual verbatim responses and to ensure that no information was lost during the data collection process. This facilitated verbatim transcriptions, which is a key aspect of data analysis, rather than focusing on the researcher’s notes alone (Polit & Beck 2017:557).

### Data analysis

Data from the study were analysed by the principal researcher under the guidance of the two co-researchers. The data analysis process commenced immediately after the one-on-one in-depth interviews, in order to identify gaps and inform further data collection. The researcher listened to the audio recordings and read through the transcriptions several times. This was made for her to familiarise herself with the data and to write down any impressions: the process that Maree (2016:111) refers to as ‘memoing’. Qualitative content analysis was followed to analyse the collected data (Maree 2016:111). Codes were grouped to form categories and then similar categories formed themes. The type of coding used during data analysis is termed ‘open coding’. Through open coding, the researchers were able to thoroughly examine the data for similarities and differences (Maree 2016:114; Polit & Beck 2017:558). Emerging themes were considered as the findings of this study. The principal researcher did peer debriefing with research supervisors or co-researchers and reached consensus with regard to the emerging themes.

### Framework of quality criteria

The quality of this study was assured by using Whittermore and colleagues’ framework (cited in Polit & Beck 2017:585). The framework uses four primary criteria that are essential to all qualitative enquiries, namely credibility, authenticity, criticality and integrity. Credibility addresses the issue of how congruent the findings are with reality (Maree 2016:123). In this study, credibility was ensured through persistent observation, collecting data until saturation was reached, ensuring triangulation and peer debriefing. Persistent observation was made when the researcher focused on conversations and aspects that were related to feedback in clinical settings. Peer debriefing was made through reviews and discussions with the two research supervisors, who are experts in qualitative research and health science education.

Authenticity is the extent to which researchers fairly and faithfully show ranges of realities (Polit & Beck 2017:585). It was ensured through verbatim transcription of all interviews and writing the report in a way that readers will understand students’ perceptions of feedback in clinical settings. Criticality refers to the researchers’ critical appraisal of every decision made throughout the research process (Polit & Beck 2017:586). Criticality was ensured by analysing the available evidence on the research process, feedback in clinical settings and deep application of critical thinking in order to make decisions related to the study. According to Shaw and Satalkar (2018:79–93), integrity is being transparent, honest and objective. It generally addressed the importance of sticking to the research question and avoiding bias in data interpretation. In this study, the researcher engaged in self-reflection, self-scrutiny and documentation of all steps throughout the study, which further enhanced the criticality and integrity of the study. The two criteria were observed together because they are strongly interrelated (Polit & Beck 2017:586). In addition, a pre-test of the interview guide was performed with two nursing students who also met the sampling criteria. The findings from the pre-test were transcribed and analysed.

### Ethical considerations

Ethical clearance (S16/04/072) was obtained from the Health Research Ethics Committee of Stellenbosch University. Written permission to interview students was granted by the research committee of University of Namibia as well as the office of the coordinator of the Health Training Centre. In addition, the study received ethical approval by the Office of the Health Ministry Permanent Secretary via the Research Unit (protocol reference number 17/3/3). Informed consent was obtained from participants by signing a copy of the participant information sheet together with a consent form, which also included permission to audio record and transcribe data thereafter. This was performed prior to data collection. No coercion or any form of bribe was used to recruit participants. In addition, the participants were informed of their rights to withdraw from the study at any stage or chose not to respond to some questions. In this study, confidentiality was maintained by ensuring that data were not linked to any participant’s name. In addition, voice recordings and hard-copy data were stored in a locked cupboard, whilst data in a soft-copy version were stored on a laptop protected by a password. Transcribed data were given code numbers and no names were identified with the interview scripts.

## Findings

Eleven participants took part in the study. They were all fulltime nursing students, aged between 19 and 28 years, including seven females and four males. The analysis of the data revealed four themes, which are as follows: feedback is perceived as a teaching and learning process in clinical settings; participants perceived the different nature of feedback in clinical settings; participants perceived personal and interpersonal implications of feedback in clinical settings and there are strategies to improve feedback in clinical settings. The themes and categories that were generated from the study are presented in Table 1.

**TABLE 1:** Themes and categories generated from the study.

No.	Themes	Categories
Theme 1	Feedback is perceived as a teaching and learning process in clinical settings.	Feedback is perceived to enhance learning in clinical settings. Feedback is perceived as a teaching method in clinical settings.
Theme 2	Perceived different nature of feedback in clinical settings.	Positive feedback perceived in clinical settings. Nursing students perceive to receive group feedback in clinical settings. Perceived communication-related challenges in clinical settings. Written feedback as perceived by nursing students in clinical settings. Perceived lack of feedback uniformities in clinical settings.
Theme 3	Perceived personal and interpersonal implications of feedback in clinical settings.	Perceived personal implications of feedback. Feedback is perceived to enhance interpersonal relations in clinical settings. Feedback is perceived to evoke emotional reactions in clinical settings.
Theme 4	Strategies to improve feedback in clinical settings.	Establishment of procedures and guidelines on feedback. Coordination of nursing students’ training in clinical settings because of perceived lack of teamwork, scheduled feedback time and a focal person for teaching.

### Theme 1: Feedback is perceived as a teaching and learning process in clinical settings

Participants in this study were aware that feedback is a teaching and learning process that occurs in clinical settings. Participants reported on the influence of feedback on learning and also how it is utilised as a teaching pedagogy.

#### Category 1.1: Feedback is perceived to enhance learning in clinical settings

The participants indicated that feedback helps to improve their knowledge and skills, which means that it enhances learning. Knowledge and skills are critical components in nursing education because they indicate that a student is competent in a specific area. Through feedback, students think of an idea as to whether the knowledge possessed is true and relevant to their learning objectives and that skills performance is up to expected standards. In nursing, different procedures have specific and standard operating guidelines that are followed by nurses in clinical settings. Therefore, these procedures serve as a guiding tool during daily practices. As novices to the nursing profession, students are taught to perform procedures according to these guidelines. This was mentioned in the interviews:

‘Feedback helps me improve my skills and knowledge in clinical areas, especially in the areas of midwifery and general nursing science.’ (Participant 8, 26-year-old female, Nursing Diploma student)

‘It [feedback] helps you fix your mistakes.’ (Participant 5, 20-year-old female, Nursing Degree student)

Participants from the current study further urged that feedback helps students to be engaged in the learning process by giving their input and coming up with solutions together with the feedback provider. This was mentioned by two participants:

‘Some registered nurses allow you to give input on what you have learned in the department.’ (Participant 1, 20-year-old male, Nursing Degree student)

‘Sometimes they ask you to give your views and then you come up with a solution together.’ (Participant 8, 26-year-old female, Nursing Diploma student)

#### Category 1.2: Feedback is perceived as a teaching method in clinical settings

From the participants’ responses, it is evident that feedback is perceived by nursing students as a teaching method in clinical settings. This means that feedback time is also a teaching moment where registered nurses demonstrate short practical skills or teach students correct ways to perform nursing procedures. Participants reflected:

‘For example if I took long to cut the umbilical cord or if I cut it at the wrong site, during feedback session the sister show me, you are supposed to cut like this (demonstrating skill)….’ (Participant 7, 25-year-old female, Nursing Diploma student)

### Theme 2: Perceived different nature of feedback in clinical settings

Positive, group and written feedback was cited in this study as the nature of feedback received by nursing students in Keetmanshoop district. Moreover, communication-related issues and a lack of uniformity in feedback were also perceived.

#### Category 2.1: Positive feedback perceived in clinical settings

Participants claimed that they received positive feedback after performing procedures in clinical settings. The positive feedback is also given in the form of positive comments made by the registered nurses at the end of their shifts in the clinical settings. Additionally, participants indicated that they gain useful, detailed, critique and valuable information from the nurse mentors about their performance in clinical settings. This was interpreted as positive feedback. The following statements were mentioned:

‘[… *F*]or example they can tell you … I like your eagerness, I like the way you relate theory with practice.’ (Participant 6, 23-year-old male, Nursing Diploma student)

‘One gets corrections, critiques and valuable information on what might have been done wrong or left out so that one does not repeat the same mistakes. I can also add that we really get detailed and useful comments.’ (Participant 10, 20-year-old female, Nursing Certificate student)

#### Category 2.2: Nursing students perceive to receive group feedback in clinical settings

Participants indicated that nurses in clinical settings like to give general feedback to a group of nursing students. When nursing students are given group feedback, the feedback providers call all of them to gather at one place and then convey information. In the context of the current study, students may gather to receive feedback according to their levels of study, training institutions or programme of study. This was perceived as a hindrance to learning in clinical settings as they did not really learn more about their individual shortcomings. In most cases, feedback given to a group does not highlight the performance of a specific student but rather summarise the message intended for all students allocated in a specific department. This was indicated by the following quotes:

‘Sometimes they can tell the whole group that most of you still do not know how to record vital signs … such statements are too vague because you don’t know if you need to improve or if you are doing it right.’ (Participant 6, 26-year-old male, Nursing Diploma student)

‘There is no one- on- one feedback, feedback seems to address the group rather than the individual, it’s not really important to me because it doesn’t address me personally.’ (Participant 9, 22-year-old male, Nursing Certificate student)

#### Category 2.3: Perceived communication-related issues in clinical settings

Participants perceived that there are communication-related challenges pertaining to feedback provided and received in clinical settings. Participants identified a lack of interaction between the feedback providers and the students as a common practice in clinical settings, which means that the provider dominates the entire session without accepting the input from the feedback receiver. Participants mentioned that they were not invited to participate and were afraid of questioning or giving their personal opinions as the feedback provider might not help them again. These views indicate that nursing students were not open to requests for further information because of fear that it might result in mistreatment in the future. This was expressed as follows:

‘After observing how you perform a procedure, the sister tells you how you performed and gives your book back, sometimes you want to ask but you can’t since you’re not invited to comment.’ (Participant 8, 26-year-old female, Diploma Nursing student)

Moreover, it was also revealed that feedback was conveyed in the form of non-verbal communication such as cues showing that performance was up to the expected standards. It appears that nursing students are able to notice feedback conveyed to them in other forms, rather than verbal and written communication only. This was indicated by the following quote:

‘They don’t really use words to tell how you’re performing, sometimes it manifests in trust, if they notice that you can perform a certain procedure without any assistance, they will always delegate that task to you.’ (Participant 4, 21-year-old female, Degree Nursing student)

#### Category 2.4: Written feedback as perceived by nursing students in clinical settings

In degree, diploma and certificate nursing training programmes, students receive an evaluation form that is completed by nurses from the clinical settings. The form is returned to the training institution and in most cases, it is delivered as a confidential document, particularly when negative aspects concerning the student’s performances are reported. However, the evaluation may also be given to the student for hand delivering to the lecturers or clinical instructors at the training institutions. The students may open and read the evaluation form when it is handed to them for delivery to the training institution. In this study, participants recognised the evaluation forms that they received from their training institutions as part of the feedback they received in clinical settings, although the forms were not directed at them. This was indicated as follows:

‘When we go out for practical, we get two evaluation forms, the registered nurses give feedback on our performance and give it back to you to take to the training centre.’ (Participant 1, 20-year-old male, Degree Nursing student)

‘The registered nurses write comments in our evaluation forms, although it’s not meant for us, I get time to read it before handing it to the lecturers.’ (Participant 3, 22-year-old female, Degree Nursing student)

#### Category 2.5: Perceived lack of feedback uniformities in clinical settings

Participants indicated that they rotated amongst different settings within the intermediate hospital and clinics. They noticed differences in the way and nature of feedback being given in clinical settings and these differences were also noticed by individual feedback providers. Participants further perceived that lack of uniformity was because of unavailability of standards or guidelines to follow when providing feedback. As it appears, feedback providers gave feedback without following any standard form in terms of frequency, timing or steps of feedback provision. Instead, feedback was based on their own principles. A participant expressed:

‘What I noticed is that nurses at maternity ward are stricter, they give more detailed feedback, maybe because the maternity department is more critical.’ (Participant 2, 21-year-old male, Nursing Degree student)

‘There seems to be no procedures to follow when giving feedback because everyone does it differently.’ (Participant 3, 22-year-old female, Nursing Degree student)

### Theme 3: Perceived personal and interpersonal implications of feedback in clinical settings

Given that feedback involves communication between the provider and receiver and also that it focuses on performance of an individual student, feedback was perceived to have interpersonal implications in clinical settings. This theme is formed by three categories, described as follows:

#### Category 3.1: Perceived personal implications of feedback

Participants reported that feedback is associated with self-evaluation and self-development. It was perceived that feedback helped nursing students to engage in self-reflection and further boosted qualities such as self-confidence, self-motivation, self-esteem and a sense of personal satisfaction. These are all part of self-development:

‘Feedback aids in reflection on the part of the student.’ (Participant 10, 20-year-old female, Nursing certificate student)

‘Feedback is very important because it helps increase students’ confidence, self-motivation, self-esteem and sense of personal satisfaction.’ (Participant 6, 20-year-old male, Nursing Diploma student)

Feedback-seeking behaviour also emerged as a code from the study findings. Participants revealed that when nursing students are not given feedback in clinical settings, they ask for it from their supervisors. This was mentioned as:

‘If like me if I don’t get feedback and I see that sister is not busy I ask if we can talk about my performance. But not all sisters take it positive, some tell you they are resting.’ (Participant 11, 21-year-old female, Nursing Certificate student)

#### Category 3.2: Feedback is perceived to enhance interpersonal relations in clinical settings

Learning in clinical settings is facilitated by the good interpersonal skills that are required between students and mentors, students and peers/fellow students, students and patients or clients they are serving and also between students and other members of the healthcare team. The participants indicated that feedback helped to build strong relationships between them, their mentors and supervisors in clinical settings. Participants mentioned:

‘Students have good relation with nurses who give them feedback, we are free to communicate to them and ask for advice …’ (Participant 6, 23-year-old male, Nursing Diploma student)

‘Feedback builds a good work relationship between nurses and students.’ (Participant 4, 21-year-old female, Nursing Degree student)

#### Category 3.3: Feedback is perceived to evoke emotional reactions

In this study, feedback was perceived to evoke emotional reactions because of how students reacted after receiving feedback. This implies that when positive feedback is received, it is followed by positive reactions from the receiver and vice versa. However, participants stressed that despite negative feedback and feedback conveyed inhumanely, nursing students try to maintain a positive mindset and reactions. Another emotional reaction experienced by the participants was that some feedback providers used the time to give feedback as their chance to express their negative emotions and feedback was provided in a harsh manner. It was mentioned that:

‘Some nurses are very rude, but we can’t fight with them. Maybe the nurse is angry at that moment; sometimes students have to remain calm.’ (Participant 8, 26-year-old female, Nursing Diploma student)

‘Sometimes after receiving feedback, especially if the sisters shout at me, I go to a quit room to cry. But if sister was happy and smiling during feedback, I smile back.’ (Participant 7, 25-year-old female, Nursing Diploma student)

### Theme 4: Strategies to improve feedback in clinical settings

During the interviews with participants, they gave suggestions of how feedback in clinical settings may be improved. The suggestions called for collaborative approach, by including feedback providers, management of the district hospital and the training institutions. The two categories that formed this theme are procedures and guidelines on feedback, as well as coordination of students’ training in clinical settings.

#### Category 4.1: Establishment of procedures and guidelines on feedback

As a strategy to improve nursing students’ performance and monitor the impact of feedback, participants recommended that follow-ups are conducted based on feedback in the clinical settings. The following is an example of a quote from the students:

‘I am suggesting that nurses … or educators… (long silent) should follow up on students to see if they’re improving based on their feedback or they can delegate another person to follow up.’ (Participant 2, 21-year-old male, Nursing Degree student)

Participants revealed that there are no feedback guidelines adopted in the clinical settings in the Keetmanshoop District. Findings indicated that there were discrepancies in the way feedback was conveyed, indicating a lack of a common feedback framework, which is why the findings recommended the development of a guideline. The following statement supports this view:

‘The way they give us feedback sometimes (shaking head) … I cannot blame them because maybe they were not trained or guided to give feedback. They teach so well but seem to have no idea on how to give students feedback. I am requesting experts in the field to write standard guidelines to be followed when giving us feedback.’ (Participant 2, 21-year-old male, Nursing Degree student)

#### Category 4.2: Coordination of nursing students’ training in clinical settings because of perceived lack of teamwork, scheduled feedback time and a focal person for teaching

Participants felt that feedback in clinical settings can be improved by incorporating it into the departmental daily routine. This can be made by allocating teaching and learning time daily. Another suggestion from participants was for improved teamwork between training institutions and clinical facilities. This was provoked by the fact that students received conflicting feedback because there seemed to be a lack of teamwork between the training institutions and the clinical settings. Finally, participants perceived that the availability of a focal person or training nurse for each department would improve feedback for nursing students. This was suggested because nurse mentors were occupied with nursing care duties and did only clinical teaching when there were no clinical-related activities to take care of. The following was mentioned by participants:

‘One nurse can tell you your performance is excellent and the next moment one can tell you that you don’t know anything and that’s not how the procedure is supposed to be performed. What is worse, your lecturer did not even teach you about it, now you don’t know which one to follow, they should work together (shouting)!’ (Participant 4, 21-year-old male, Nursing Degree student)

‘It’s important for the government to employ training nurses who are responsible for students in each department.’ (Participant 6, 23-year-old male, Nursing Diploma student)

## Discussion

The purpose of this study was to explore nursing students’ perceptions of the feedback they received in clinical settings, at the district hospital. In this study, nursing students perceived feedback to be part of the teaching and learning process in clinical settings, which is given in different formats. They also perceived that there are individuals and interpersonal implications of feedback in clinical settings. During interviews, the participants offered some strategies to improve feedback in clinical settings.

The findings on feedback as part of teaching and learning processes are in accordance with studies found during the literature control. A study conducted by Dawson et al. (2019:25–36) at two Australian universities reported that students perceive the purpose of feedback as improvement their understanding and performance in general. Also, a review study conducted in North Carolina by Jug, Jiang and Bean (2019:244–250) on giving and receiving feedback reported future improvement as one of the perceived purposes of feedback amongst students. In addition, a study conducted in the United Kingdom revealed that students perceived feedback as contributing unremittingly to the process of learning and improving performance (Deeley et al. 2019:385–405). However, they warned that it is important for students to use feedback effectively for it to positively contribute to learning. Therefore, Mcfadzien (2015:16–18) indicated that when students receive feedback, they should first notice and trust that providers are willing to help them and then use it to advance performance. Mcfadzien (2015:16–18) continued that teachers are no longer the transmitter of all knowledge and there is a need to engage students in the learning process through student-centred approaches. Feedback supports the involvement of students in the learning process via bidirectional conversations between the feedback provider and receiver (Jug et al. 2019:244–250). The initial problem identified in the current study’s setting was that feedback did not help nursing students improve or complete their practical registers, as it was too general. However, the findings revealed the significant role of feedback in the teaching and learning process as it helps nursing students to enhance their knowledge and skills, by providing students an opportunity to correct their mistakes.

Feedback is equally important to both students (feedback receiver) and e teachers or educators (feedback providers) as it is a fundamental practice to both teaching and learning (Mcfadzien 2015:16–18). Effective feedback can offer information to teachers that can be used to advance teaching strategies (Al-Bashir et al. 2016:38–41). Therefore, it helps providers to understand how teaching practice can be enhanced (AITSL 2017:n.p.). It is recommended for teachers to integrate feedback processes with their pedagogical knowledge to effectively respond to students’ learning needs (Heitink et al. 2016:50–62). The relation of feedback and teaching found in the literature, as explained, is consistent with what was mentioned by the participants in the current study. The participants in this study perceived the feedback process as a teaching pedagogy, which is used as a platform to impart knowledge and skills in clinical settings through their demonstration of skills during the feedback session.

The current study revealed that nursing students obtain positive feedback, which is evident in positive comments, as well as detailed, useful critique and valuable feedback received in clinical settings. These findings oppose initial problems observed in the current study’s setting, namely, that feedback received by nursing students was experienced as a barrier to completing their practical workbooks as it was too general. Moreover, the findings of the current study align with Dawson et al. (2019:25–36), who revealed that comments made on the students’ work were positive, supportive, constructive and encouraging. This is contrary to Carless (2019:705–714) who discovered that comments received by students were unsupportive, limited and directed at task level.

In the setting of the current study, group feedback was a common practice. This could be because of the large number of health science students in clinical settings and the shortage of nursing staff. The nursing staff play a dual role, meaning that they are responsible for patient care and clinical teaching of students in their units. This results in nursing staff focusing more on patient care and limited time is spent on the students. Hardavella et al. (2017:327–333) emphasised that feedback should be individualised and given in private because students perceived group feedback as a criticism, which may negatively affect relationships. In addition, students feel insulted and undermined as a result of feedback given in groups. Conflicting results were documented by Deeley et al. (2019:385–405), who revealed that students find it beneficial to receive group feedback. Nevertheless, Hardavella et al. (2017:327–333) indicated that feedback providers may give group feedback, but this should be addressed to the whole group rather than targeting an individual within the group.

Feedback can be referred to as a process that encompasses the communication of information followed by responses to such communication (Mandhane et al. 2015:1868–1873). This implies that feedback is a form of communication that is equally important to both the sender and the receiver, in warranting that communication takes place and is in effect (Winstone et al. 2017:17–37). Therefore, the communication in the feedback process should be mutual (Hardavella et al. 2017:327–333). In the current study, feedback did not involve the nursing students as there were no interactions between the provider and receiver of feedback. The feedback was not interactive, therefore, not effective as it did not incorporate the students’ input, especially on the way forward, and how shortcomings may be improved to fill the gap between reality and the desired goal. Although it was not mentioned by participants in the current study, it is known that students benefit little from feedback when there is no interaction with the feedback giver (Winstone et al. 2017:17–37).

In general, written feedback is preferred and perceived to be beneficial to students, in comparison to other forms of feedback (Deeley et al. 2019:385–405). This is more related to the fact that it is possible to recycle written comments for later use (Al-Bashir et al. 2016:38–41). Although participants in the current study recognised evaluation forms as part of the feedback they received, evaluation and feedback differ from each other. However, in reality, there is overlap between evaluation and feedback. Jug et al. (2019:244–250) asserted that feedback is a formative assessment, whilst evaluation is a form of summative assessment. Besides that, feedback may be a more informal learning tool, whilst evaluation provides cumulative performance reports. There is no evidence of a study that examined or explored the use of and experiences of using evaluation forms as feedback in clinical settings.

According to Jug et al. (2019:244–250), feedback can be associated with the interpersonal relationship between students and educators. Moreover, students’ reaction to feedback is influenced by their own personal characteristics (Carless & Boud 2018:1315–1325). These findings concur with the current study, which revealed that feedback in clinical settings had effects on personal and interpersonal relations, that is, for both nursing students and the feedback providers. Furthermore, findings of the current study revealed that after feedback sessions, nursing students engaged in activities for self-improvement such as self-reflection and self-evaluation. This concurs with Al-Bashir et al. (2016:38–41) who indicated that feedback promotes the process of reflection or self-assessment in learning. Other findings indicate that feedback has a positive impact on students’ personal and professional development (Hardavella et al. 2017:327–333) as students evaluate their own actions and judge performances.

Although the current study was conducted to explore nursing students’ perceptions on feedback received in clinical settings, the findings also indicated that nursing students portrayed the ability to seek feedback from registered nurses. According to Carless (2019:705–714), feedback-seeking behaviours are normally observed from ambitious and pro-active students who are eager to learn and adopt a deep approach to learning. Hardavella et al. (2017:327–333) emphasised that students should ask for feedback if they have not received any after their performances. Although literature revealed that students in clinical settings receive feedback from patients and peers (Houghton 2016:41–49; Pedram et al. 2020:1–10), in the context of the current study, nursing students only indicated to receive feedback from the registered and enrolled nurses, as well as clinical instructors and lecturers from the training institutions. This could mean that patients are not given an opportunity to provide feedback to students, especially on issues like compassionate care, respect and the ability to treat them with dignity, which are fairly judged from the eye of the service receiver. Feedback not being given by peers could be because of no incorporation of peer teaching, assessment and observation in clinical settings of the current study.

The participants in the current study alluded that feedback is conveyed to them in a negative way. This includes rude mannerisms that are accompanied by offensive and negative comments. Moreover, a positive attribute interpreted from nursing students is that, despite feedback being conveyed to them in a negative way, they remained calm and took the situation in a positive way. If feedback was conveyed to a student in a positive way, it will be taken positively and will be accompanied by happiness together with feelings of accomplishment. When feedback is perceived by the student as criticism, the responses will be denial, anger and blaming. To avoid these reactions, feedback providers are warned against giving feedback when they are angry (Qureshi 2017:243–248).

The purpose of feedback is to help learners develop their future performance or learning strategies (Carless 2019:705–714). The development for future performance requires an action plan on changes that should take place and it, therefore, needs follow-ups to be conducted. Feedback is not a once off activity; it is a process coupled with a sequence of actions that are cyclical in nature. Therefore, the participants in the current study suggested the need for follow-ups on feedback. This is because they perceived that feedback is given and then no follow-up takes place to determine what has been improved and what the student is still struggling to achieve. Historically, educators have rectified students’ work without any theory of feedback involved (Boud & Molloy 2013:689–712). In addition, feedback was purely accepted as information provided by teachers to students about their work. As the field of health professional education advances, the experts have developed and documented models, tips, techniques and guidelines to adopt as frameworks to help educators convey feedback and students receive it. In the current study’s setting, nursing students perceived that there are uniformities in the nature and manner of how feedback is conveyed to the nursing students. These findings are contrary to those of Deeley et al. (2019:385–405), who asserted that students noted inconsistency in the way feedback was conveyed by different staff. This recommendation on the development of procedures and guidelines for feedback is crucial, considering that registered nurses are not trained as clinical educators and a large number of lecturers, tutors and clinical instructors are appointed in their positions because of qualifications in their discipline that are, however, not education-related.

Because of the dual roles of registered nurses (teaching and patient care), the participants suggested the allocation of teaching time during clinical blocks and an allocation of a teaching and learning focal person in the units as a coordination measure. The allocation of teaching and learning time was supported by Al-Bashir et al. (2016:38–41), who documented that it is necessary to choose the right moment to give feedback. In the current study, it was perceived that the teaching and learning time would be ideal moment to give feedback. There is no documented evidence about allocating the teaching and learning focal person as there are lecturers, clinical instructors and preceptors who guide the students in most health science programmes. However, institutions are stressing the importance of feedback through training, workshops and courses directed at individuals who guide students (Qureshi 2017:243–248). A well-coordinated teaching and learning process in clinical settings may help to address problems such as teamwork between training institutions and clinical settings, which was also perceived to be lacking in the context of current study.

The findings from the current study may have implications related to learning and teaching in clinical settings. This is because feedback is one of the most dominant influences on learning and nursing students’ achievement in clinical settings. The findings of the current study may improve learning in clinical settings in such a way that nursing students receive positive and written feedback, which has personal and interpersonal implications. Feedback was further found to have the potential to influence personal development and therefore may close the gap between desired and real performance. Furthermore, the study exposed areas that needed improvement, such as the development of guidelines and procedures for feedback providers to help in the process of feedback in clinical settings, as well as the coordination of activities. This may improve teaching and learning in clinical settings as it may lead to effective and constructive feedback.

### Limitations of the study

The study focused on nursing students’ perceptions of feedback they received in clinical settings; therefore, this does not include the perceptions of feedback providers.

### Recommendations

Based on the findings of the study, the following recommendations for nursing education, nursing management and future research are made:

- Nurse educators should develop a framework, model or guidelines to be used by staff members who supervise students in clinical settings. This may reduce inconsistency and provide guidance in the provision of feedback. In addition, it may provide tips on how to integrate feedback in their day-to-day activities.
- As part of the nursing students’ preparation for clinical practice, they should be made aware of the importance of feedback and receive tips to be more receptive to feedback.
- The research findings revealed communication-related issues such as a lack of interaction and non-verbal methods of conveying feedback. The feedback providers should be encouraged to engage with nursing students in the process and make use of other forms of feedback, rather than non-verbal methods to avoid the risk of misunderstandings.
- Further research may explore the feedback providers’ perspectives of feedback in clinical settings, as the current study only explored the perceptions of nursing students.

## Conclusion

Feedback is one of the basic elements that should be present in educational strategies utilised in clinical settings as it helps to close the gap between real and desired performance. The purpose of this study was to explore nursing students’ perceptions of the feedback they received in clinical settings, at the district hospital. The findings indicated that feedback is perceived to be part of the teaching and learning process in clinical settings. Moreover, different forms were used to convey feedback to nursing students in clinical settings. Strategies were suggested by nursing students for the improvement of feedback in clinical settings. The recommendations made based on the findings were directed towards nursing education, nursing management and further research. This will ensure that a framework is in place to guide feedback providers in the provision of feedback, nursing students are prepared to receive feedback and the perceptions of feedback providers in clinical settings are explored.

## References

[CIT0001] Abraham, R.M. & Singaram, V.S., 2016, ‘Third-year medical students’ and clinical teachers’ perceptions of formative assessment feedback in the simulated clinical setting’, *African Journal of Health Professions Education* 8(1), 121–125. 10.7196/AJHPE.2016.v8i1.769

[CIT0002] Adamson, E., King, L., Foy, L., McLeod, M., Traynor, J., Watson, W. et al., 2018, ‘Feedback in clinical practice: Enhancing the students’ experience through action research’, *Nurse Education in Practice* 31, 48–53. 10.1016/j.nepr.2018.04.012 29753252

[CIT0003] AITSL, 2017, *Spotlight: Reframing feedback to improve teaching and learning*, viewed 05 December 2017, from https://www.aitsl.edu.au/docs/default-source/research-evidence/spotlight/spotlight-feedback.pdf?sfvrsn=cb2eec3c_12.

[CIT0004] Al-Bashir, M., Kabir, R. & Rahman, I., 2016, ‘The value and effectiveness of feedback in improving students’ learning and professionalizing teaching in higher education’, *Journal of Education and Practice* 7(16), 38–41.

[CIT0005] Alfehaid, L.S., Qotineh, A., Alsuhebany, N., Alharbi, S. & Almodaimegh, H., 2018, ‘The perceptions and attitudes of undergraduate healthcare sciences students of feedback: A qualitative study’, *Health Professions Education* 4(3), 186–197. 10.1016/j.hpe.2018.03.002

[CIT0006] Allen, L. & Molloy, E., 2017, ‘The influence of a preceptor-student “daily feedback tool” on clinical feedback practices in nursing education: A qualitative study’, *Nurse Education Today* 49, 57–62. 10.1016/j.nedt.2016.11.009 27888784

[CIT0007] Boud, D. & Molloy, E., 2013, ‘Rethinking models of feedback for learning: The challenge of design’, *Assesment & Evaluation in Higher Education* 38(6), 689–712. 10.1080/02602938.2012.691462

[CIT0008] Burgess, A. & Mellis, C., 2015, ‘Feedback and assessment for clinical placements: achieving the right balance’, *Advances in Medical Education and Practice* 19(6), 373–381. 10.2147/AMEP.S77890 PMC4445314 26056511

[CIT0009] Carless, D., 2019, ‘Feedback loops and the longer-term: towards feedback spirals’, *Assessment and Evaluation in Higher Education* 44(5), 705–714. 10.1080/02602938.2018.1531108

[CIT0010] Carless, D. & Boud, D., 2018, ‘The development of student feedback literacy: Enabling uptake of feedback’, *Assessment and Evaluation in Higher Education* 43(8), 1315–1325. 10.1080/02602938.2018.1463354

[CIT0011] Creswell, J., 2014a, *Educational research: Planning, conducting and evaluating qualitative and quantitative research*, Pearson, London.

[CIT0012] Creswell, J., 2014b, *Research design: Qualitative, quantitative and mixed methods approaches*, Sage, Los Angeles, CA.

[CIT0013] Dasila, K., Veer, B., Ponchitra, R., Divya, K.Y. & Singh, S., 2016, ‘Perceptions of nursing students on clinical teaching behaviors of teaching faculty: Correlational survey design’, *IOSR Journal of Nursing and Health Science* 5(5, version 6), 37–41.

[CIT0014] Dawson, P., Henderson, M., Mahoney, P., Phillips, M., Ryan, T., Boud, D. et al., 2019, ‘What makes for effective feedback: Staff and student perspectives’, *Assessment and Evaluation in Higher Education* 44(1), 25–36. 10.1080/02602938.2018.1467877

[CIT0015] Deeley, S.J., Fischbacher-Smith, M., Karadzhov, D. & Koristashevskaya, E., 2019, ‘Exploring the “wicked” problem of student dissatisfaction with assessment and feedback in higher education’, *Higher Education Pedagogie* 4(1), 385–405. 10.1080/23752696.2019.1644659

[CIT0016] Dobrowolska, B., Mcgonagle, I., Jackson, C., Kane, R., Cabrera, E., Cooney-Miner, D. et al., 2015, ‘Clinical practice models in nursing education: Implication for students’ mobility’, *International Nursing Review* 62(1), 36–46. 10.1111/inr.12162 25559068

[CIT0017] Fowler, P. & Wilford, B., 2016, ‘Formative feedback in the clinical practice setting: What are the perceptions of student radiographers?’, *Radiography* 22(1), e16–e24. 10.1016/j.radi.2015.03.005

[CIT0018] Gaberson, K.B., Oermann, M. & Shellenbarger, T., 2015, *Clinical teaching strategies in nursing*, Springer, New York.

[CIT0019] González-García, M., Lana, A., Zurrón-Madera, P., Valcárcel-álvarez, Y. & Fernández-Feito, A., 2020, ‘Nursing students’ experiences of clinical practices in emergency and intensive care unit’, *International Journal of Environmental Research and Public Health* 17(16), 1–14. 10.3390/ijerph17165686 PMC7459869 32781646

[CIT0020] Hardavella, G., Aamli-Gaagnat, A., Saad, N., Rousalova, I. & Sreter, K.B., 2017, ‘How to give and receive feedback effectively’, *Breathe* 13(4), 327–333. 10.1183/20734735.009917 29209427 PMC5709796

[CIT0021] Heitink, M., Van der Kleij, F., Veldkamp, B., Schildkamp, K. & Kippers, W., 2016, ‘A systematic review of prerequisites for implementing assessment for learning in classroom practice’, *Educational Research Review* 17, 50–62. 10.1016/j.edurev.2015.12.002

[CIT0022] Houghton, T., 2016, ‘Assessment and accountability: Part 1 – Assessment’, *Nursing Standard* 30(38), 41–49. 10.7748/ns.30.38.41.s44 27191451

[CIT0023] Johnson, C.E., Keating, J.L., Farlie, M.K., Kent, F., Leech, M. & Molloy, E.K., 2019, ‘Educators’ behaviours during feedback in authentic clinical practice settings: An observational study and systematic analysis’, *BMC Medical Education* 19(1), 1–11. 10.1186/s12909-019-1524-z 31046776 PMC6498493

[CIT0024] Jug, R., Jiang, X.S. & Bean, S.M., 2019, ‘Giving and receiving effective feedback: A review article and how-to guide’, *Archives of Pathology and Laboratory Medicine* 143(2), 244–250. 10.5858/arpa.2018-0058-RA 30102068

[CIT0025] Kalyani, M.N., Jamshidi, N., Molazem, Z., Torabizadeh, C. & Sharif, F., 2019, ‘How do nursing students experience the clinical learning environment and respond to their experiences? A qualitative study’, *BMJ Open* 9(7), 1–8. 10.1136/bmjopen-2018-028052 PMC6661598 31350243

[CIT0026] Mandhane, N., Ansari, S., Shaikh, T. & Deolekar, S., 2015, ‘Positive feedback: A tool for quality education in field of medicine’, *International Journal of Research in Medical Sciences* 3(8), 1868–1873. 10.18203/2320-6012.ijrms20150293

[CIT0027] Maree, K., 2016, *First steps in research*, Van Schaick, Pretoria.

[CIT0028] Mcfadzien, N., 2015, ‘Why is effective feedback so critical in teaching and learning?’, *Journal of Initial Teacher Inquiry* 1, 16–18. 10.26021/844

[CIT0029] Pedram, K., Brooks, M.N., Marcelo, C., Kurbanova, N., Paletta-Hobbs, L., Garber, A.M. et al., 2020, ‘Peer observations: Enhancing bedside clinical teaching behaviors’, *Cureus* 12(2), 1–10. 10.7759/cureus.7076 PMC7093940 32226677

[CIT0030] Polit, D. & Beck, C., 2017, *Nursing research: Generating and assessing evidence for nursing practice*, Wolters Kluwer, Philadelphia, PA.

[CIT0031] Pront, L. & McNeill, L., 2019, ‘Nursing students’ perceptions of a clinical learning assessment activity: “Linking the puzzle pieces of theory to practice”’, *Nurse Education in Practice* 36, 85–90. 10.1016/j.nepr.2019.03.008 30889469

[CIT0032] Qureshi, N.S., 2017, ‘Giving effective feedback in medical education’, *The Obstetrician & Gynaecologist* 19(3), 243–248. 10.1111/tog.12391

[CIT0033] Rajeswaran, L., 2017, ‘Clinical experiences of nursing students at a selected institute of health sciences in Botswana’, *Health Science Journal* 10(6), 1–6. 10.21767/1791-809x.1000471

[CIT0034] Shaw, D. & Satalkar, P., 2018, ‘Researchers’ interpretations of research integrity: A qualitative study’, *Accountability in Research* 25(2), 79–93. 10.1080/08989621.2017.1413940 29291621

[CIT0035] Sultan, A.S. & Khan, M.A.M., 2017, ‘Feedback in a clinical setting: A way forward to enhance student’s learning through constructive feedback’, *Journal of the Pakistan Medical Association* 67(7), 1078–1084. 10.1136/bjsm.2007.035881 28770891

[CIT0036] Walsh, L.J., Anstey, A.J. & Tracey, A.M., 2018, ‘Student perceptions of faculty feedback following medication errors – A descriptive study’, *Nurse Education in Practice* 33, 10–16. 10.1016/j.nepr.2018.08.017 30216803

[CIT0037] Weinstein, D.F., 2015, ‘Feedback in clinical education: Untying the Gordian knot’, *Academic Medicine* 90(5), 559–561. 10.1097/ACM.0000000000000559 25406602

[CIT0038] Winstone, N.E., Nash, R.A., Parker, M. & Rowntree, J., 2017, ‘Supporting learners’ agentic engagement with feedback: A systematic review and a taxonomy of recipience processes’, *Educational Psychologist* 52(1), 17–37. 10.1080/00461520.2016.1207538

